# Caractéristiques épidémio-cliniques de la tuberculose génitale chez la femme tunisienne: une série de 47 cas

**DOI:** 10.11604/pamj.2018.30.71.14479

**Published:** 2018-05-29

**Authors:** Souheil Zayet, Aida Berriche, Lamia Ammari, Mariem Razgallah, Rim Abdelmalek, Mohamed Khrouf, Badreddine Kilani, Hanène Tiouiri Benaissa

**Affiliations:** 1 Service des Maladies Infectieuses, Hôpital la Rabta, Tunis, Tunisie; 2 Université Tunis El Manar, Faculté de Médecine de Tunis,Tunisie; 3 Service de Pharmacie, Centre de Greffe de Moelle Osseuse, Tunis, Tunisie; 4 Service de Gynécologie Obstétrique, Hôpital Aziza Othmana, Tunis, Tunisie

**Keywords:** Tuberculose génitale, femme, cœlioscopie diagnostique, quadrithérapie, Genital tuberculosis, woman, diagnostic coelioscopy, quadritherapy

## Abstract

L'objet est de relever les caractéristiques épidémio-cliniques, diagnostiques, thérapeutiques et évolutives de la tuberculose génitale (TG) chez la femme en Tunisie. Nous avons mené une étude rétrospective, descriptive au service des maladies infectieuses de l'hôpital la Rabta de Tunis, sur une période de 15 ans et demi (janvier 2000-juin 2014). Nous avons inclus toutes les patientes hospitalisées pour une tuberculose génitale. 47 cas ont été colligés. L'âge moyen était de 42,2 ans. Dix-huit femmes étaient d'origine rurale. Un comptage tuberculeux a été retrouvé dans cinq cas. Pour tous les cas, le début était insidieux. Un ou plusieurs signes d'imprégnation tuberculeuse ont été rapportés dans 23 cas. L'intradermoréaction (IDR) à la tuberculine réalisée chez 35 femmes (74,8%), était positive dans 26 cas (74%). Trente-neuf patientes (83%) avaient eu une exploration radiologique par une échographie et / ou une tomodensitométrie (TDM) abdomino-pelvienne. Une cœlioscopie diagnostique a été réalisée dans 37 cas (75,5%). L'examen Anatomopathologique a permis de confirmer le diagnostic de TG dans 42 cas (89,3%) en retrouvant un granulome épithéloïde et giganto-cellulaire. Nous avons relevé 21 cas de TG isolée, alors que les 26 autres avaient une atteinte péritonéale associée. Toutes les patientes ont reçu une antibiothérapie spécifique associant l'Isoniazide, la Rifampicine, le Pyrazinamide et l'Ethamubutol pour une durée moyenne de 12 mois. Aucune patiente n'a reçu de corticothérapie et aucune chirurgie secondaire n'a été indiquée. L'évolution était favorable dans 39 cas, 8 patientes ont été perdues de vue. La TG est une pathologie rare qui ne représente que 0,5% de la tuberculose extra-pulmonaire, mais d'un grand polymorphisme clinique. La confirmation diagnostique est difficile et repose sur l'étude bactériologique et/ou histologique. Le diagnostic doit être évoqué devant toute symptomatologie abdomino-pelvienne trainante, devant une stérilité chez la femme, associé à un contexte épidémio-clinique évocateur.

## Abstract (en)

This study aimed to identify the epidemio-clinic, diagnostic, therapeutic and evolutionary features of genital tuberculosis (GT) among Tunisian women. We conducted a retrospective, descriptive study in the Department of Infectious Diseases at the La Rabta Hospital, Tunisia, over a period of 15 and a half years (January 2000 - June 2014). All patients hospitalized for genital TB were included in the study. The study focused on 47 cases. The average age of patients was 42.2 years. Eighteen women were from rural areas. Tuberculous contact was found in five cases. In all cases, the onset was insidious. Twenty-three patients showed one or several signs of TB infection. Tuberculin intradermal reaction (IDR) test was performed in 35 women (74.8%), it was positive in 26 cases (74%). Thirty-nine patients (83%) had undergone radiological examination using abdomino-pelvis ultrasound and/or CT scan. Diagnostic coelioscopy was performed in 37 cases (75.5%). Anatomopathological examination helped to confirm the diagnosis of GT in 42 cases (89.3%), showing epithelioid and giant-cell granuloma. We identified 21 cases of isolated GT, the remaining 26 cases had peritoneal involvement. All patients received specific antibiotic therapy combining isoniazid, rifampicin, pyrazinamide and ethamubutol with an average treatment duration of 12 months. No patient received corticosteroids or secondary surgery. Patients’ outcome was favorable in 39 cases, 8 patients were lost to follow-up. Genital tuberculosis is rare, representing only 0.5% of extra-pulmonary tuberculosis, but it accounts for a high prevalence of clinical polymorphism. Diagnostic confirmation is difficult and it is based on bacteriological and/or histological examinations. Diagnosis should be suspected in patients with chronic abdominopelvic symptoms, in women with infertility associated with suggestive epidemioclinical manifestations.

## Introduction

La tuberculose génitale (TG) de la femme fait partie des formes rares et peu connues des tuberculoses extra-pulmonaires. Il s'agit d'une pathologie peu fréquente dans les pays développés, touchant essentiellement les femmes issues de milieu défavorisé. Elle atteint aussi bien les femmes jeunes que les femmes ménopausées. Dans la majorité des cas, l´agent causal est le *Mycobacterium tuberculosis* et plus rarement *Mycobacterium bovis* . La TG occasionne chez la femme des lésions tubaires et utérines à l'origine d'une infertilité. Elle est souvent de découverte fortuite et toute sa gravité est liée à ses conséquences sur la fertilité. La TG de la femme peut se présenter sous forme de masses tubo-ovariennes avec atteinte péritonéale, ressemblant à un tableau de pathologie tumorale. La certitude diagnostique est apportée par la microbiologie et /ou l'histologie. C'est la biopsie des annexes par voie cœlioscopique qui permet souvent, de confirmer le diagnostic, évitant ainsi un geste radical d'exérèse chirurgicale et permettant d'instaurer un traitement médical.

## Méthodes

Pour réaliser ce travail, nous avons mené une étude rétrospective, descriptive au service des maladies infectieuses de l'hôpital la Rabta de Tunis sur une période de 15 ans et demi, soit de janvier 2000 à juin 2014. Nous avons inclus toutes les femmes hospitalisées pour une tuberculose génitale confirmée histologiquement et/ou bactériologiquement, ou ayant un faisceau d'arguments épidémio-cliniques, radiologiques et thérapeutiques en faveur de ce diagnostic. Toutes les données ont été recueillies à partir des dossiers des malades. Nous avons relevé les données épidémiologiques (âge, sexe, origine géographique, profession, antécédents de tuberculose, consommation de lait cru, vaccination par le BCG, présence d'une immunodépression), cliniques (signes d'imprégnation tuberculeuse, signes fonctionnels, signes physiques), paracliniques (biologiques, bactériologiques, radiologiques et Anatomopathologiques), thérapeutiques et évolutives. Le diagnostic de TG a été retenu sur un faisceau d'arguments bactériologiques et/ou histologiques : mise en évidence du bacille acido-alcoolo-résistant (BAAR) dans le liquide d'ascite ou sur broyat de la biopsie, ou présence de granulome épithéloïde et giganto-cellulaire avec nécrose caséeuse sur la pièce de la biopsie.

## Résultats

**Figure 1:**
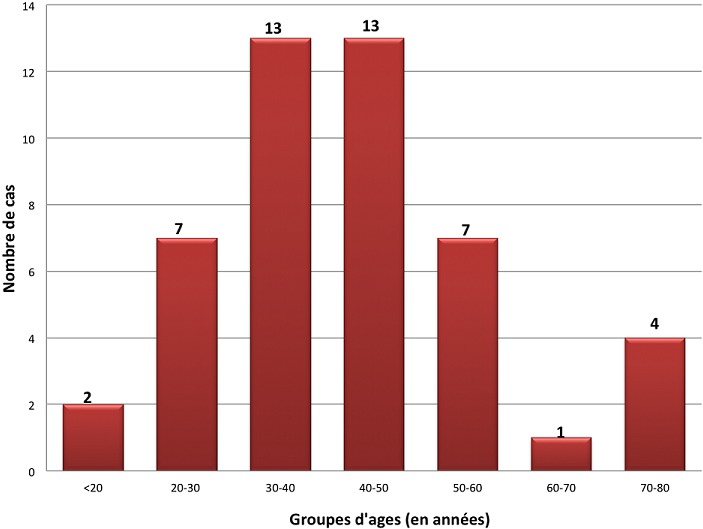
Tranches d'âge des femmes ayant une tuberculose génitale

**Figure 2:**
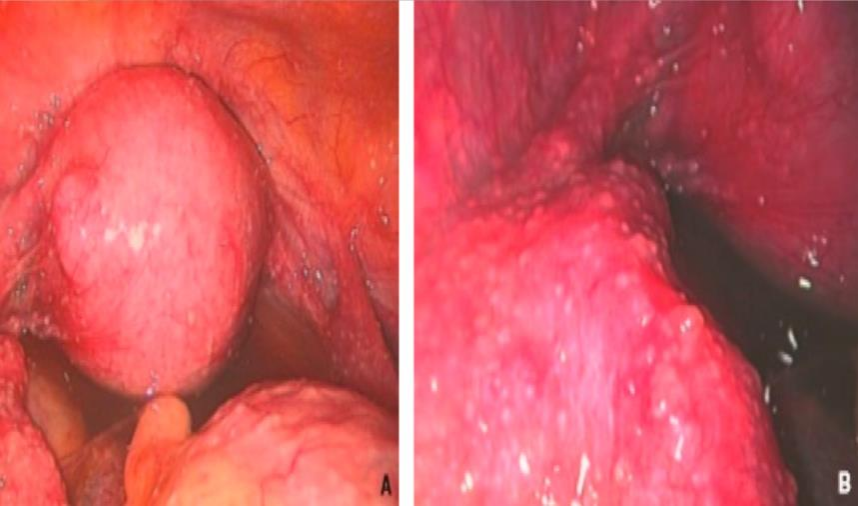
Tuberculose génitale de la femme au stade subaigu vue en cœlioscopie A): aspect inflammatoire du péritoine avec hyper vascularisation et œdème, nombreux granulations recouvrant le péritoine, épanchement minime jaunâtre dans le cul-de-sac de Douglas; B): vue rapprochée sur l’annexe gauche : nombreux granulations blanchâtres

Nous avons colligé 47 femmes. L'âge moyen était de 42,2 ans (extrêmes: 18-76 ans). La tranche d'âge la plus touchée est celle des 30-50 ans (Figure 1). 18 femmes étaient d'origine rurale (38,3%) et 36 étaient des femmes au foyer (76,6%). Un comptage tuberculeux a été retrouvé dans 5 cas (10,6%), un antécédent personnel de tuberculose dans quatre cas (8,5%) et une consommation de produits laitiers non pasteurisés dans 5 cas (10,6%). La cicatrice de vaccination par le BCG n'a été recherchée que dans 5 cas, et était présente pour 3 d´entre eux. La sérologie VIH n'a pas été systématiquement demandée, mais aucune patiente n'était connue infectée par le VIH. Concernant les comorbidités, 6 patientes étaient hypertendues, 3 diabétiques, une avait une β-thallassémie, une était suivie pour néoplasie du sein, une autre pour hépatite C chronique et une dernière pour épilepsie. Concernant les antécédents gynécologiques, 3 patientes avaient une aménorrhée primaire (6,4%), 9 une aménorrhée secondaire (19,9%), 12 patientes avaient une stérilité primaire (25,5%) et 4 une stérilité secondaire (8,5%). Pour tous les cas, le début était insidieux. Le délai moyen de consultation était de 11 mois et 5 jours (extrêmes: 1mois-6ans). Le tableau clinique était polymorphe. Un ou plusieurs signes d'imprégnation tuberculeuse ont été rapportés chez 23 patientes (48,9%). L'amaigrissement a été rapporté dans 19 cas (40,4%), l'anorexie dans 18 cas (38,3%), la fièvre, les sueurs nocturnes et l'asthénie dans 17 cas chacune (36,2%). 21 femmes avaient des douleurs pelviennes (44,7%), 16 femmes des douleurs abdominales (34%), 10 femmes avaient un ballonnement abdominal (21,3%) et 10 des métrorragies (21,3%). 9 femmes avaient une matité abdominale, évoquant une ascite (19,1%). Une intradermoréaction (IDR) à la tuberculine a été réalisée chez 35 femmes (74,8%). Elle était positive dans 26 cas (74%). Une ponction d'ascite a été réalisée dans 2 cas. L'aspect du liquide était jaune citrin dans les 2 cas, et exsudatif avec un taux moyen d'albumine de 29,7 g/l. L'étude cytologique a montré une pléiocytose moyenne de 85 éléments/mm^3^ (extrêmes: 7-163/mm^3^). L'examen direct à la recherche de BAAR ainsi que la culture de BK étaient négatives dans les 2 cas. La radiographie du thorax, faite pour toutes les patientes était normales dans 95,7% des cas. Une imagerie abdomino-pelvienne a été réalisée chez 39 patientes par une échographie (n = 33) et/ou une TDM abdomino-pelvienne (n = 6). L'anomalie la plus fréquemment retrouvée était une masse ovarienne, et ce dans 19 cas (40,4%) (Figure 2). Les données de l'échographie et de la TDM sont résumées dans les Tableau 1 et Tableau 2. Une cœlioscopie diagnostique a été réalisée dans 37 cas (78%). Une ou plusieurs biopsies per-opératoires ont été réalisées. Les principales lésions macroscopiques étaient des granulations blanchâtres dans 22 cas (59,4%), une atteinte tubaire dans 7 cas (18,9%) et masse ovarienne dans 7 cas également (18,9%), avec des associations possibles. Chaque patiente avait une ou plusieurs lésions. Les données de la coelioscopie sont résumées dans le Tableau 3. L'examen anatomopatholgique a permis de confirmer le diagnostic de TG dans 89,4% des cas (n = 42). 3 biopsies étaient perdues après leur réalisation ou non concluantes à l'examen histologique. Les sièges des biopsies sont résumés dans le Tableau 4. Un granulome épithéloïde et giganto-cellulaire a été retrouvé chez 100% des patientes dont la pièce de biopsie a été étudiée, associé à une nécrose caséeuse dans 78,5% des cas. Le délai moyen du diagnostic était de 11 mois et 24 jours (extrêmes: 1 mois - 5 ans). Au terme des données cliniques et des explorations radiologiques, nous avons relevé 21 cas de TG isolée (44,7%). Une atteinte péritonéale était associée dans 26 cas (55,3%). D'autres localisations associées ont été retrouvées dans 21,2% des cas. Les différentes localisations associées sont résumées dans le Tableau 5. Un traitement anti-tuberculeux a été instauré dans tous les cas. Il s'agissait d'une quadrithérapie associant l'Isoniazide, la Rifampicine, le Pyrazinamide et l'Ethambutol pendant deux mois relayée par une bithérapie par l'Isoniazide et la Rifampicine, pour une durée totale moyenne de 12 mois (extrêmes: 7 mois - 32 mois). L'observance au traitement était bonne dans 93,6% des cas. Un ou plusieurs effets indésirables ont été notés chez 11 patientes. Il s'agissait d'une intolérance digestive à type de pyrosis dans deux cas, de paresthésies dans deux cas, d'une toxidermie dans un cas, d'une hyperuricémie symptomatique dans quatre cas, de cytolyse et de cholestase dans un cas chacune. Aucune patiente n'a reçu de corticothérapie et aucune chirurgie secondaire n'a été indiquée. La durée moyenne d'hospitalisation était de 16 jours (extrêmes: 4-36 jours). L'évolution clinique était favorable dans 83% des cas (n = 39), avec disparition des signes fonctionnels et une prise de poids. Les huit autres patientes étaient perdues de vue.

**Tableau 1:** Aspects échographiques

Aspect échographique	Nombre	Pourcentage (%)
Masse ovarienne	19	40,4
Fibrome	9	19,1
Masse abdominale	3	9
Ascite	3	9
Echographie normale	7	21,2
Nombre total	33	70,2

**Tableau 2:** Aspects tomodensitométriques

Aspect scannographique	Nombre	Pourcentage (%)
Ascite	2	4,2
Masse	2	4,2
Abcès	1	2,1
Ganglions	1	2,1
Nombre total	6	12,8

**Tableau 3:** Aspects de la cœlioscopie diagnostique

Aspect de la cœlioscopie	Nombre	Pourcentage (%)
Granulations	22	59,4
Adhérences	11	29,7
Atteinte tubaire	7	18,9
Masse ovarienne	7	18,9
Ascite	5	13,5
Nombre total	37	78

**Tableau 4:** Siège de la biopsie

Siège de la biopsie	Nombre	Pourcentage (%)
Annexectomie	19	42,2
Granulations	16	35,5
Hystérectomie	8	17,7
Curetage Biopsique de l’Endomètre	6	13,3
Trompe	2	4,4
Nombre total	45	95,7

**Tableau 5:** Localisations associées

Localisation associée	Nombre	Pourcentage (%)
Ganglionnaire	3	6,4
Urinaire	2	4,2
Pleurale	1	2,1
Ganglionnaire et pleurale	1	2,1
Digestive	1	2,1
Intestinale	1	2,1
Spondylodiscite	1	2,1
Total	10	21,2

## Discussion

La tuberculose dans le monde reste toujours un sujet d'actualité. En 2015, 10,4 millions de personnes ont contracté cette maladie et 1,8 million en sont décédées [1]. Dans les pays en voie de développement tel que le nôtre, cette pathologie reste fortement endémique. La forme extra-pulmonaire, souvent paucibacillaire, connait un regain d'intérêt en raison d'une augmentation inexpliquée de leur fréquence relative atteignant 20 à 40% selon les séries [2]. En particulier, la localisation génitale féminine demeure sous-estimée et peu citée dans la littérature, ce qui explique le retard du diagnostic [3]. A notre connaissance, il s'agit de la deuxième série tunisienne de TG féminine, après celle de Hammami et al. portant sur 22 cas et dans laquelle les auteurs avaient conclu à une prévalence de 6 à 10% de l'ensemble des localisations tuberculeuses [4]. La TG est toujours secondaire et succède soit à une dissémination par voie hématogène provenant d'un autre foyer tuberculeux (en premier lieu le poumon) soit à une contamination par voie lymphatique à partir des ganglions pelviens. Une infection primaire reste possible mais exceptionnelle [5]; Weinstein a rapporté de rares cas de TG secondaires à une inoculation directe par transmission sexuelle entre partenaires [6]. La tuberculose pelvienne touche de façon prédominante la femme jeune, en pleine activité génitale. La moyenne d'âge dans notre série, qui est de 42 ans, concorde avec les données de la littérature [4,5,7], avec une prédominance des cas pour la tranche d'âge 30-50 ans. Les formes tardives déclarées en péri ou post-ménopause sont dues le plus souvent à une longue période de latence de la maladie [8], comme c'est le cas de 5 de nos patientes âgées de plus que 60 ans et dont 2 étaient diagnostiquées suite une cœlioscopie avec biopsies d'une masse utérine. Cette affection touche classiquement les femmes de faible niveau économique [4,7,9], alors que la majorité de nos patientes sont de niveau économique moyen. Aucun signe fonctionnel ou physique n'est pathognomonique de la TG ; cette pathologie est caractérisée par un grand polymorphisme clinique [9]. Si auparavant, les formes classiques ascitiques et les pelvipéritonites étaient les plus fréquentes, les formes actuelles sont plutôt pauci-symptomatiques et souvent découvertes à la suite d'une infertilité primaire ou secondaire (44%). Comme dans les principales publications, 30 à 50 % des cas sont diagnostiqués à l'occasion de bilans de stérilité. En effet, la stérilité primaire est le motif de consultation le plus fréquent dans les principales publications [10,11].

**Figure 3:**
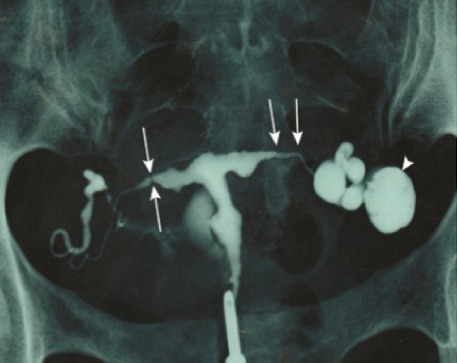
Hystérosalpingographie (Images de soustractions latérales et bilatérales de la cavité utérine donnant un aspect en «T» (flèches). Images de constrictions tubaires des deux côtés. Obstruction tubaire distale bilatérale: phimosis à droite et hydrosalpinx gauche (tête de flèche))

Dans une étude indienne récemment publiée colligeant plus de 30000 femmes stériles, l'étiologie tuberculeuse a été retrouvée dans 15% des cas [12], ce qui justifie la recherche systématique d'une TG chez toute femme stérile. Les troubles du cycle à type d'aménorrhée primaire ou secondaire sont un autre motif de consultation, retrouvés dans 5 à 20% des cas [13]. L'aménorrhée est souvent en rapport avec la présence de synéchies utérines, à l'origine de la diminution de réceptivité de l'endomètre, ou due à une anovulation secondaire aux adhérences péritonéales. Dans notre étude, seulement 3 patientes avaient une aménorrhée primaire (6,4%) alors que 9 avaient une aménorrhée secondaire (19,1%). L'examen clinique est souvent pauvre et peu contributif. Toutefois, il peut mettre en évidence une masse latéro-utérine à la palpation bi-manuelle ou retrouver une ascite [14]. Le bilan biologique est également d'un intérêt limité et non spécifique. La radiographie pulmonaire doit être réalisée pour rechercher une localisation pulmonaire primaire. Dans la série de Ravelosoa à Madagascar, 8 femmes sur 11 ayant une TG avaient une symptomatologie respiratoire concomitante en rapport avec une localisation pulmonaire [7]. L'urographie intraveineuse est nécessaire en raison de la fréquence de la tuberculose urinaire associée [15]. L'hystérosalpingographie (HSG) est un examen d'orientation de choix en cas de doute diagnostique au cours de la TG [16,17]. Dans la série de Sharma et al, l'HSG a permis d'orienter le diagnostic dans plus de la moitié des cas montrant une irrégularité des contours tubaires dans 18% des cas, des synéchies réalisant un aspect typiquement en doigt de gant dans 17% des cas et une opacification de l'endocol ou de l'isthme seulement dans 18% des cas [18]. Un aspect de sténose des trompes, d'images d'abcès ou d'hydrosalpinx ont été décrit dans notre série (Figure 3). Une extravasation veineuse et lymphatique du produit de contraste est possible [16]. Si d'autres lésions génitales sont associées, la cœlioscopie peut présenter un intérêt majeur pour le diagnostic (Figure 2) et pour la recherche d'autres localisations de la tuberculose. Le diagnostic de certitude de la TG est obtenu par la mise en évidence du bacille acido-alcoolo-résistant, soit à l'examen direct, soit après mise en culture des prélèvements pathologiques, qui sont obtenus par curetage biopsique endométrial, ou par laparoscopie voire laparotomie [19]. Les techniques d'amplification nucléique ont considérablement facilité le diagnostic de certaines formes de tuberculose extrapulmonaire. La détermination rapide de la présence du génome bactérien par polymerase chain reaction (PCR) constitue actuellement un progrès récent dans le diagnostic de la TG [12,20], avec une spécificité et une sensibilité dépassant les 60% dans certaines études [21]. L'examen histologique d'une biopsie de l'endomètre, de lésion péritonéale ou de masse tubo-ovarienne reste l'examen clé pour confirmer le diagnostic, tout en sachant que la nécrose caséeuse peut manquer dans d'authentiques tuberculoses. La négativité de cet examen n'exclut pas le diagnostic et doit au contraire inciter à multiplier les prélèvements. Dans notre étude, la tuberculose génitale était confirmée par l'étude histologique dans 89,4% des cas.

Le traitement est avant tout médical par antibiothérapie associant l´Isoniazide, la Rifampicine, le Pyrazinamide et l'Ethambutol pendant 2 mois relayée par une bithérapie par isoniazide et rifampicine pour une durée totale de 12 mois [9], avec une surveillance clinique et paraclinique régulière. Une chirurgie complémentaire ne se justifie qu'en présence de lésions volumineuses réagissant peu ou pas au traitement anti-tuberculeux ou dans le cadre d'un désir de grossesse. La chirurgie devrait être réalisée au moins 6 semaines après le début de la chimiothérapie anti bacillaire qui réduit le risque des complications peropératoires. Une cure de synéchie par hystéroscopie peut être proposée malgré un haut risque de perforations [22]. Depuis l'avènement des techniques de procréation médicalement assistée (PMA), la Fécondation In Vitro (FIV) considéré comme le gold standard de la prise en charge de l'infertilité tubaire permet d'obtenir de bons résultats, en cas d'infertilité [23]. Cependant les taux de grossesse chez les femmes atteintes de TG restent bas du fait des lésions endométriales observées [23], ainsi que la baisse du pool ovarien, décrite par plusieurs auteurs [24]. Cette technique reste toutefois difficile d'accès dans les pays en voie de développement. Face à la gravité des séquelles gynécologiques, souvent majorées par le retard diagnostique et la sous-estimation de cette pathologie, il nous paraît essentiel de renforcer la prévention par la vaccination systématique par le BCG avant l´âge de 15 ans avec la croissance de l'incidence des formes extrapulmonaires de la tuberculose à un âge précoce [25]. Certes, cette étude présente quelques limites; il s'agit d'une série non randomisée rétrospective. Secondairement, le diagnostic a été porté essentiellement sur les données de l'histologie sans recours aux méthodes de biologie moléculaire, en particulier à la PCR dans la détection du BK du fait que cet outil n'est toujours pas disponible dans notre structure hospitalière.

## Conclusion

La tuberculose génitale est une pathologie rare, d'un grand polymorphisme clinique dont l'incidence réelle reste probablement sous-estimée. La confirmation diagnostique est difficile et repose sur l'étude bactériologique et/ou histologique. Elle doit être évoquée devant tout tableau abdominal ou pelvien d'évolution traînante, ou lors de l'investigation d'une stérilité chez la femme associée à un contexte épidémio-clinique évocateur. L'importance d'un dépistage précoce et systématique chez les femmes présentant des troubles du cycle menstruel ou une stérilité doit être soulignée, particulièrement dans les pays à forte incidence de tuberculose.

### Etat des connaissances actuelle sur le sujet

- La tuberculose génitale chez la femme est une forme extra-pulmonaire de la tuberculose rare avec un grand polymorphisme clinique;
- La stérilité secondaire devrait toujours faire penser à ce diagnostic surtout en présence d'un contexte épidémiologique évocateur;
- La confirmation diagnostique de la tuberculose génitale semble être toujours difficile, survenant de façon tardive.

### Contribution de notre étude à la connaissance

- Il s'agit de la plus grande série tunisienne descriptive de cette forme de tuberculose extra-pulmonaire;
- Notre série met le point sur l'association des deux formes de tuberculose péritonéale et génitale chez la femme et incite à une recherche systématique de localisation génitale en cas d'atteinte péritonéale;
- Les tableaux de ce papier constituent la partie la plus utile apportant des éléments nouveaux dans la littérature.

## Conflits d’intérêts

Les auteurs ne déclarent aucun conflit d'intérêts.
